# Deubiquitination and Activation of the NLRP3 Inflammasome by UCHL5 in HCV-Infected Cells

**DOI:** 10.1128/spectrum.00755-21

**Published:** 2021-08-25

**Authors:** Akshaya Ramachandran, Binod Kumar, Gulam Waris, David Everly

**Affiliations:** a Center for Cancer Cell Biology, Immunology, and Infection, Chicago Medical School, Rosalind Franklin University of Medicine and Science, North Chicago, Illinois, USA; Texas A&M University

**Keywords:** hepatitis C virus, NLRP3, IL-1β, UCHL5, deubiquitination, inflammation

## Abstract

Chronic hepatitis C virus (HCV) infection induces liver inflammation that can lead to fibrosis, cirrhosis, and hepatocellular carcinoma (HCC). Inflammation is the outcome of the action of proinflammatory cytokines and chemokines, including interleukin-1 beta (IL-1β) and tumor necrosis factor alpha. Mature IL-1β production and secretion are facilitated by active inflammasome complexes, including the NACHT-LRR pyrin domain-containing protein 3 (NLRP3) inflammasome. Our study shows that the NLRP3 inflammasome is activated in HCV-infected hepatocytes and that the activation is regulated by posttranslational modifications. NLRP3 is modified by lysine-63 ubiquitin chains in hepatocytes and is deubiquitinated during HCV infection. Inhibition of deubiquitinases (DUBs) with chemical inhibitors or blocking UCHL5 DUB expression with small interfering RNA (siRNA) abrogated NLRP3 inflammasome assembly and activation. Inhibition of inflammasome deubiquitination was correlated with a reduction in IL-1β maturation, decrease in HCV protein expression, and reduction in release of HCV from the cells. Together, this study suggests that HCV-induced activation of the NLRP3 inflammasome through posttranslational modification is crucial for the HCV life cycle and pathogenesis.

**IMPORTANCE** HCV infection induces inflammation leading to fibrosis, cirrhosis, and cancer. The current study identifies the mechanisms leading to the activation of the NLRP3 inflammasome in hepatocytes, which is an important site of viral replication. Deubiquitination of NLRP3 by UCHL5 is required for inflammasome activation. Inhibition of deubiquitination blocks NLRP3 inflammasome activation and IL-1β maturation and also decreases HCV replication, suggesting the importance of the NLRP3 inflammasome in inflammation as well as other signaling pathways.

## INTRODUCTION

Hepatitis C virus (HCV) infection is known to cause liver disease beginning with liver inflammation that can progress to liver cirrhosis, fibrosis, and hepatocellular carcinoma in chronically infected patients (1). The positive-sense, single-stranded RNA genome of HCV is translated to a single 3,000-amino-acid polyprotein, which is cleaved by host and viral proteases to form structural (HCV core, E1, and E2) and nonstructural (p7, NS2, NS3, NS4a, NS4b, NS5a, and NS5b) proteins. These structural and nonstructural viral proteins form a ribonucleoprotein complex to replicate the viral genome and assemble virions to complete the virus life cycle (2). Although a number of treatments, including interferon therapy and direct-acting antivirals, have been discovered that can cure HCV infection, there is still a need to understand the biology of the virus and its pathogenesis. This understanding can help in identifying treatment options for HCV-induced liver diseases and inflammatory liver diseases due to other causes (3).

Inflammation is the response of the host following injury as well as infections caused by bacteria or viruses. Initiation of the inflammatory response occurs in response to sensing of molecules, such as pathogen-associated molecular patterns (PAMPs) or danger-associated molecular patterns (DAMPs). Viral infections are sensed by PAMPs and DAMPs through Toll-like receptors (TLRs), retinoic acid-inducible gene-I-like receptors (RLRs), and protein kinase R (PKR), which result in production of interferon and inflammatory molecules. Specifically, inflammation precedes liver disease progression after HCV infection (3, 4). A number of studies have identified that inflammatory factors such as interleukin-1 beta (IL-1β), chemokine ligand 9 (CXCL9), chemokine ligand 10 (CXCL10), chemokine ligand 11 (CXCL11), chemokine ligand 5 (CCL5), and chemokine ligand 2 (CCL2) are upregulated during HCV infection, leading to inflammation (5, 6). Negash et al. showed that the production of IL-1β in Kupffer cells (liver resident macrophages) was due to activation of the NACHT-LRR pyrin domain-containing protein 3 (NLRP3) inflammasome during HCV infection (6). The NLRP3 inflammasome is a multiprotein complex that consists of the NLRP3, apoptosis-associated speck-like protein containing a CARD (ASC), and caspase-1, which act as a sensor, adaptor, and effector, respectively (7). The NLRP3 inflammasome complex formation and activation occurs through two signals. The first priming event occurs when molecules like viral RNA bind to TLR3 or TLR7, resulting in nuclear factor-kappa B (NF-κB)-mediated signaling and increased expression of inflammasome components NLRP3, ASC, and caspase-1 and expression of IL-1β precursor protein. The secondary signals, such as ATP, monosodium urate (MSU), K^+^ efflux, and mitochondrial DNA (mtDNA), cause NLRP3 inflammasome assembly and activation (7, 8). Assembly occurs through the binding of NLRP3, ASC, and caspase-1 in a multiprotein complex, leading to the autocleavage of caspase-1 into p20 and p10 active forms. Active caspase-1 can cleave precursors IL-1β, IL-18, and IL-33, resulting in their maturation and release. These cytokines help to initiate and potentiate the innate immune response and lead to the adaptive immune response.

Posttranslational modifications (PTMs) of HCV proteins as well as a number of host proteins have been observed during HCV infection (9). PTMs of host and viral proteins by phosphorylation and ubiquitination regulate the various stages of the HCV life cycle, including viral RNA translation and viral envelopment (9, 10). Activation of inflammasome proteins is tightly regulated and occurs at the transcriptional, translational, and posttranslational levels (11, 12). PTMs of proteins in the NLRP3 inflammasome, such as phosphorylation, ubiquitination, proteolytic cleavage, sumoylation, etc., can either promote or repress NLRP3 inflammasome assembly and activation (13). Specifically, assembly and activation of the NLRP3 inflammasome are controlled by phosphorylation at serine and tyrosine amino acid residues. Studies have shown that NLRP3 dephosphorylation by PTPN22 at Tyr861 leads to inflammasome activation (14). Similar modifications of NLRP3 by dephosphorylation at Ser5 by PP2A as well as JNK1-mediated phosphorylation at Ser194 have led to NLRP3 inflammasome activation (15, 16). A number of studies have also shown that NLRP3 is also regulated through ubiquitination (13). Removal of lysine-48 (K48) and lysine-63 (K63) ubiquitin chains on NLRP3 by BCCR3 leads to NLRP3 inflammasome activation, whereas K48 ubiquitination of NLRP3 by F-boxL2 prevents inflammasome activation (17, 18).

Our study focuses on understanding PTMs leading to activation of the NLRP3 inflammasome during HCV infection of human hepatoma cells. Specifically, during HCV infection, the NLRP3 inflammasome is activated and the activated inflammasome is regulated through deubiquitination. The data show that the deubiquitination of K63 ubiquitin chains on NLRP3 is mediated through deubiquitinase enzyme UCHL5, which is enzymatically active in the HCV-infected cells. The deubiquitination of NLRP3 is required for NLRP3-ASC interaction, which ultimately leads to the inflammasome formation and activation and IL-1β maturation as well as release of virus from HCV-infected cells.

## RESULTS

### HCV infection induced NLRP3 inflammasome activation.

**FIG 1:**
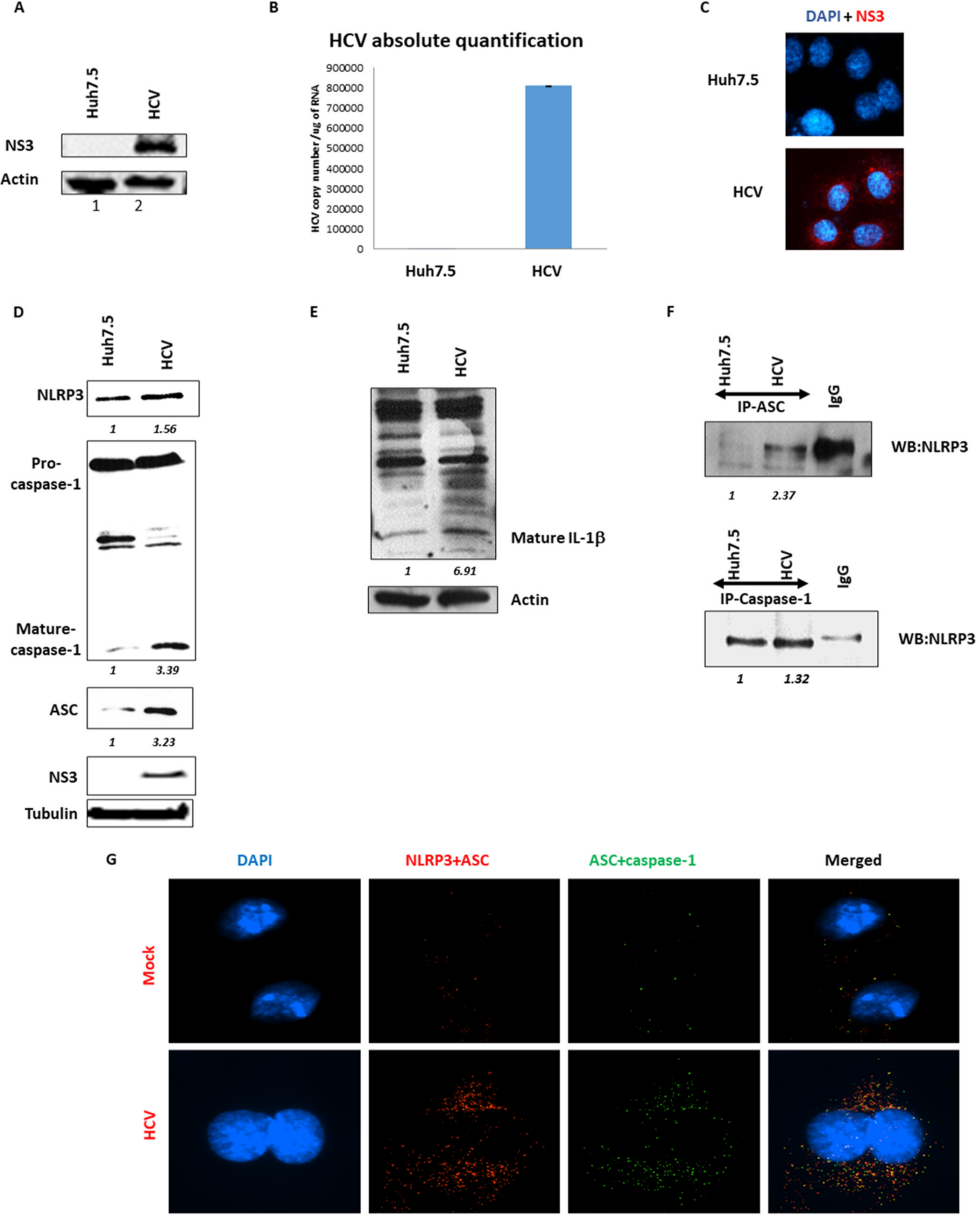
Hepatitis C virus infection induces NLRP3 inflammasome activation. (A) Huh7.5 (mock) cells were infected with HCV at 1 multiplicity of infection (MOI) and 7 days postinfection (p.i.), and the cells were harvested in RIPA buffer. Western blotting was performed using anti-HCV NS3 and actin antibody. (B) RNA extracted from mock and HCV-infected cells 7 days p.i. was quantified for HCV RNA copy number using RT-PCR. (C) Mock and HCV were fixed using 4% paraformaldehyde and permeabilized using 0.2% Triton X-100. Cells were incubated with anti-HCV NS3 antibody followed by secondary antibody (Red). DAPI is stained in blue. The imaging was performed using a Nikon i80 microscope. (D) Mock and HCV-infected cells were harvested using RIPA lysis buffer and Western blotted for NLRP3, caspase-1, ASC, HCV-NS3, and tubulin. (E) Mock and HCV-infected cells were harvested using RIPA lysis buffer and Western blotted for IL-1β and actin. (F) Mock and HCV-infected cells were harvested using NP-40 lysis buffer, and immunoprecipitation was performed using anti-ASC (top) and anti-caspase-1 (bottom) antibody. Immunoprecipitations were Western blotted for NLRP3 using anti-NLRP3 antibody. (G) Mock and day 7 p.i. HCV-infected cells were permeabilized and fixed as mentioned previously and were subjected to double proximity ligation assay using anti-NLRP3-anti-ASC (red dots) and anti-ASC-anti-caspase-1 (green dots) antibodies. The imaging was performed using a Nikon i80 microscope (scale bars are 10 μm). The merged imaged shows colocalization of the two dots in yellow. Representative images are presented from three independent experiments for panels A to F and two independent experiments for panel G.

In order to know more about the induction and posttranslational modifications of the NLRP3 inflammasome in HCV-infected human hepatocytes, Huh7.5 cells were infected with HCV and examined for inflammasome assembly (Fig. 1). HCV infection of Huh7.5 cells was confirmed by Western blotting, real-time PCR, and immunofluorescence staining. HCV viral protein NS3 expression was confirmed by Western blotting, HCV viral RNA replication was confirmed by reverse transcription-quantitative PCR (qRT-PCR), and viral protein NS3 expression was confirmed by immunofluorescence (Fig. 1A to C, respectively).

NLRP3 inflammasome formation and activation have been shown in a number of liver-associated diseases as well as HCV-infected Kupffer cells (19). To determine if the NLRP3 inflammasome complex is formed and activated in HCV-infected hepatoma cells, control and HCV-infected Huh7.5 cells were examined by Western blotting and immunoprecipitations. In HCV-infected cells, increased expression of NLRP3 and ASC, as well as activated, mature caspase-1 were observed (Fig. 1D). In addition to Western blotting, immunoprecipitation of ASC or caspase-1 pulled down the NLRP3 protein, confirming interaction of NLRP3, ASC, and caspase-1 (Fig. 1F). Finally, control and HCV-infected cells were assayed by proximity ligation assay for NLRP3+ASC and ASC+caspase-1 complexes (Fig. 1G, red and green, respectively). Both NLRP3+ASC and ASC+caspase-1 combinations had increased speckled cytoplasmic staining in HCV-infected cells, which is characteristic of NLRP3 inflammasome activation. Overlap of the red and green speckles were also observed as yellow color in the merged images, consistent with NLRP3 inflammasome assembly and activation (HCV-infected, merged panel).

The activation of the NLRP3 inflammasome results in the formation of active caspase-1, which cleaves of IL-1β into its active form. To determine if the activated NLRP3 inflammasome results in maturation of IL-1β, control and HCV-infected cells were analyzed for cleaved IL-1β by Western blotting (Fig. 1E). Cleaved IL-1β was increased nearly 7-fold in the HCV-infected cells. Together, these data indicate that the NLRP3 inflammasome is activated in HCV-infected hepatocytes, resulting in maturation of active IL-1β.

### Deubiquitination of NLRP3 during HCV infection.

**FIG 2:**
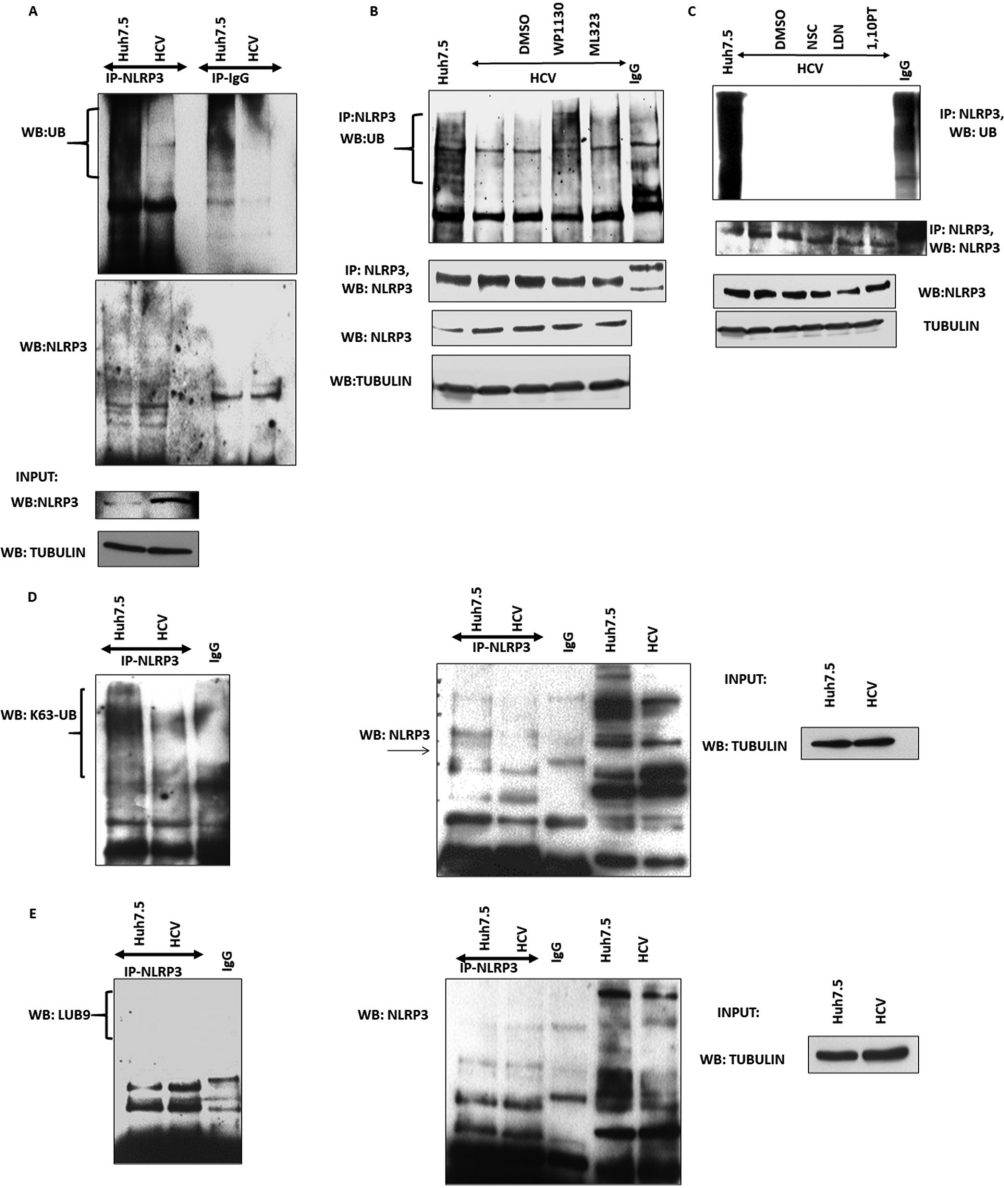
Deubiquitination of NLRP3 in HCV-infected cells. (A) Huh7.5 (mock) and HCV-infected cells at day 7 postinfection (p.i.) were lysed in ubiquitination lysis buffer. NLRP3 was immunoprecipitated (IP) using anti-NLRP3 antibody. Following IP Western blotting was performed for total ubiquitin and NLRP3. Input samples were blotted for NLRP3 and tubulin to ensure equal starting protein. (B) Mock and HCV-infected cells at day 7 p.i. were lysed in ubiquitination lysis buffer. NLRP3 was IP using anti-NLRP3 antibody and Western blotted with K63-linked ubiquitin antibody and NLRP3. Input samples were blotted for tubulin to ensure equal starting protein. (C) Mock and HCV-infected cells at day 7 p.i. were lysed in ubiquitination lysis buffer. NLRP3 was immunoprecipitated using anti-NLRP3 antibody and Western blotted with LUB9-specific ubiquitin antibody and NLRP3. Input samples were blotted for tubulin to ensure equal starting protein. (D) HCV-infected cells were treated with WP1130 (5 μM) or ML323 (5 μM) for 4 h. After treatment with inhibitors, cells were lysed in ubiquitin lysis buffer and Western blotted for NLRP3 and tubulin. The samples were also IP for NLRP3 using anti-NLRP3 antibody and Western blotted for total ubiquitin and NLRP3. (E) HCV-infected cells were treated with LDN57444 (LDN, 20 μM) for 6 h, NSC (45 μM) for 10 h, or 1,10-phenanthroline (1,10PT, 20 μM) for 4 h. After treatment with inhibitors, cells were lysed in ubiquitin lysis buffer and Western blotted and IP as in panel B. Representative images are presented from three independent experiments.

Ubiquitination is a common PTM that is known to regulate protein activation and degradation, including regulation of the NLRP3 inflammasome components. To understand the role of ubiquitination in NLRP3 inflammasome activation during HCV infection, cells were infected with HCV and the PTM of NLRP3 inflammasome components by ubiquitination were examined. In order to determine the ubiquitination status of NLRP3 during HCV infection, NLRP3 was immunoprecipitated and Western blotted for total ubiquitin. Ubiquitination of NLRP3 in uninfected cells was observed by the characteristic smear of higher-molecular-weight protein species via Western blotting. Interestingly, in HCV-infected cells, there was a drastic decrease in ubiquitinated NLRP3 (Fig. 2A). This is in contrast to the total NLRP3, which was increased in the input controls.

To determine the specific type of ubiquitin chain that is present on NLRP3, NLRP3 was immunoprecipitated, and Western blotting with antibodies specific for K63-linked ubiquitin chains and linear ubiquitin chains was performed. In uninfected cells, NLRP3 was ubiquitinated by K63-linked ubiquitin chains, and during HCV infection, there was a decrease in K63 ubiquitination (Fig. 2B). This is in contrast to the overall increase in NLRP3 levels in HCV-infected cells, indicating that the decrease in ubiquitination was not due to lower levels in NLRP3. Linear ubiquitination of NLRP3 was not observed in control or HCV-infected cells (Fig. 2C). Overall, these experimental results show that NLRP3 is modified by K63-linked ubiquitination in hepatoma cells and that K63-ubiquitination of NLRP3 is decreased during HCV infection.

### Role of deubiquitinases on NLRP3 during HCV infection.

Ubiquitination of proteins is controlled by addition of ubiquitin via ubiquitin ligases and removal of ubiquitin via deubiquitinases (DUBs). Since we observed a decrease in ubiquitinated NLRP3 during HCV infection, we focused on the role of DUBs in NLRP3 ubiquitination during HCV infection. To begin to narrow down possible DUBs that may deubiquitinate NLRP3 during HCV infection, we treated the HCV-infected cells with commercially available DUB inhibitors that have specificity for different DUBs, including WP1130, ML323, LDN57444, NSC, or 1,10-phenanthroline. NLRP3 was immunoprecipitated from mock and HCV-infected DUB inhibitor-treated cells and Western blotted for total ubiquitin. Deubiquitination of NLRP3 was prevented when the cells were treated with WP1130 during HCV infection (Fig. 2D). Increased NLRP3 ubiquitination was not observed with the other DUB inhibitors (Fig. 2D and E). We also observed that there was no effect on total NLRP3 protein expression in the DUB inhibitor-treated cells. These results suggested that the DUBs, which were inhibited by WP1130, could be induced or activated to deubiquitinate NLRP3 during HCV infection.

### Effect of UCHL5 on NLRP3 deubiquitination in HCV-infected cells.

**FIG 3:**
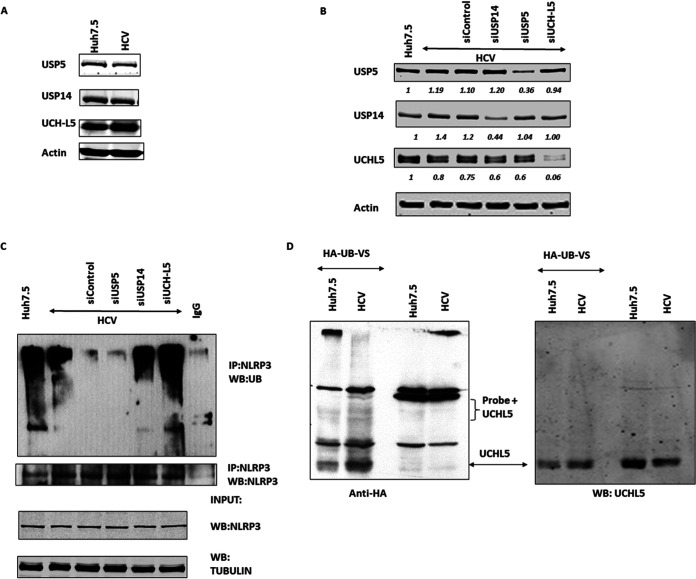
DUBs expression and their effect on NLRP3 deubiquitination. (A) Huh7.5 (mock) and HCV-infected cells were lysed in RIPA buffer 7 days postinfection (p.i.) and Western blotted for USP14, USP5, UCHL5, and actin. (B) Control, USP14, USP5, and UCHL5 siRNA was transfected at day 5 p.i. using electroporation in HCV-infected cells. Cells were lysed at day 7 p.i. and were Western blotted for USP14, USP5, and UCHL5 to confirm knockdown of target proteins. Actin was used as a loading control. (C) USP14, USP5, and UCHL5 knockdown HCV-infected cells were lysed in ubiquitin lysis buffer at day 7 p.i. and immunoprecipitated for NLRP3 using anti-NLRP3 antibody and Western blotted for total ubiquitin and NLRP3. (D) Mock and HCV-infected cells were lysed in specific buffer for DUB assay and were incubated with HA-Ub-VS probe at 37°C for 1 h. The reaction was stopped using 6× SDS loading buffer and was Western blotted with anti-HA antibody and anti-UCHL5 antibody (left and right, respectively). Representative images are presented from three independent experiments.

WP1130 is a small molecule derivative from a second class tyrphostin that selectively inhibits USP14, USP5, and UCHL5 DUBs (according to the manufacturer). To see if the decreased ubiquitinated NLRP3 was due to induction of DUB expression in HCV-infected cells, we checked the expression of deubiquitinating enzymes USP5, USP14, and UCHL5 in mock and HCV-infected cell lysates. All DUBs were expressed, and there was no significant difference in expression during HCV infection compared to control cells (Fig. 3A). This indicates that any of the three DUBs could be involved in NLRP3 deubiquitination and that the decrease in ubiquitination was not due to induced expression of the DUBs during HCV infection.

To determine which of the DUBs has a role in deubiquitinating NLRP3, the expression of each DUB was silenced via small interfering RNA (siRNA). Silencing of the individual DUBs was confirmed by Western blotting (Fig. 3B). Control, HCV-infected, and HCV-infected DUB-silenced cells were analyzed for NLRP3 ubiquitination as described above. Silencing of UCHL5 and to a lesser extent USP14 increased ubiquitinated NLRP3 (Fig. 3C). Silencing of USP5 did not increase ubiquitinated NLRP3. This suggests that UCHL5 and USP14 may be involved in removing ubiquitin chains from NLRP3. Since there were no differences in DUB expression in HCV-infected cells but silencing the DUBs did increase NLRP3 ubiquitination, this suggests that the DUBs responsible are activated without increasing their levels. To determine the activity of the DUBs, cell lysates were reacted with a probe (HA-Ub-VS) that covalently reacts with active DUBs. In addition, the covalent binding of probe to DUBs increases the molecular weight of the bound DUBs by approximately 10 kDa (the approximate molecular weight of the probe). The HA-Ub-VS probe was reacted with control or HCV-infected cell lysates, and Western blotting was performed using anti-HA antibody (Fig. 3D). Experimental results showed an increase in the number of bands that appeared in the probe + HCV lysates, suggesting that DUBs are activated during HCV infection. Additionally, several bands in the 45- to 48-kDa molecular weight range were increased, which is consistent with reacted UCHL5 (native molecular weight, 36 kDa) (Fig. 3D). The same blot was used to perform Western blotting for UCHL5, where the only bands observed are from the unbound UCHL5 (molecular weight, 36 kDa). Additionally, we also observed a faint band for probe-bound USP14 (approximately 70 kDa) but no visible increase in probe-bound USP5 (about 100 kDa). Reaction of DUBs with the probe insinuates that there are a number of DUBs activated during HCV infection, including UCHL5 and USP14. Overall, these results show that active UCHL5 is required for NLRP3 deubiquitination in HCV-infected cells and is potentially activated but not induced in HCV-infected cells.

### Effect of deubiquitination of NLRP3 by UCHL5 on NLRP3 inflammasome formation and activation.

**FIG 4:**
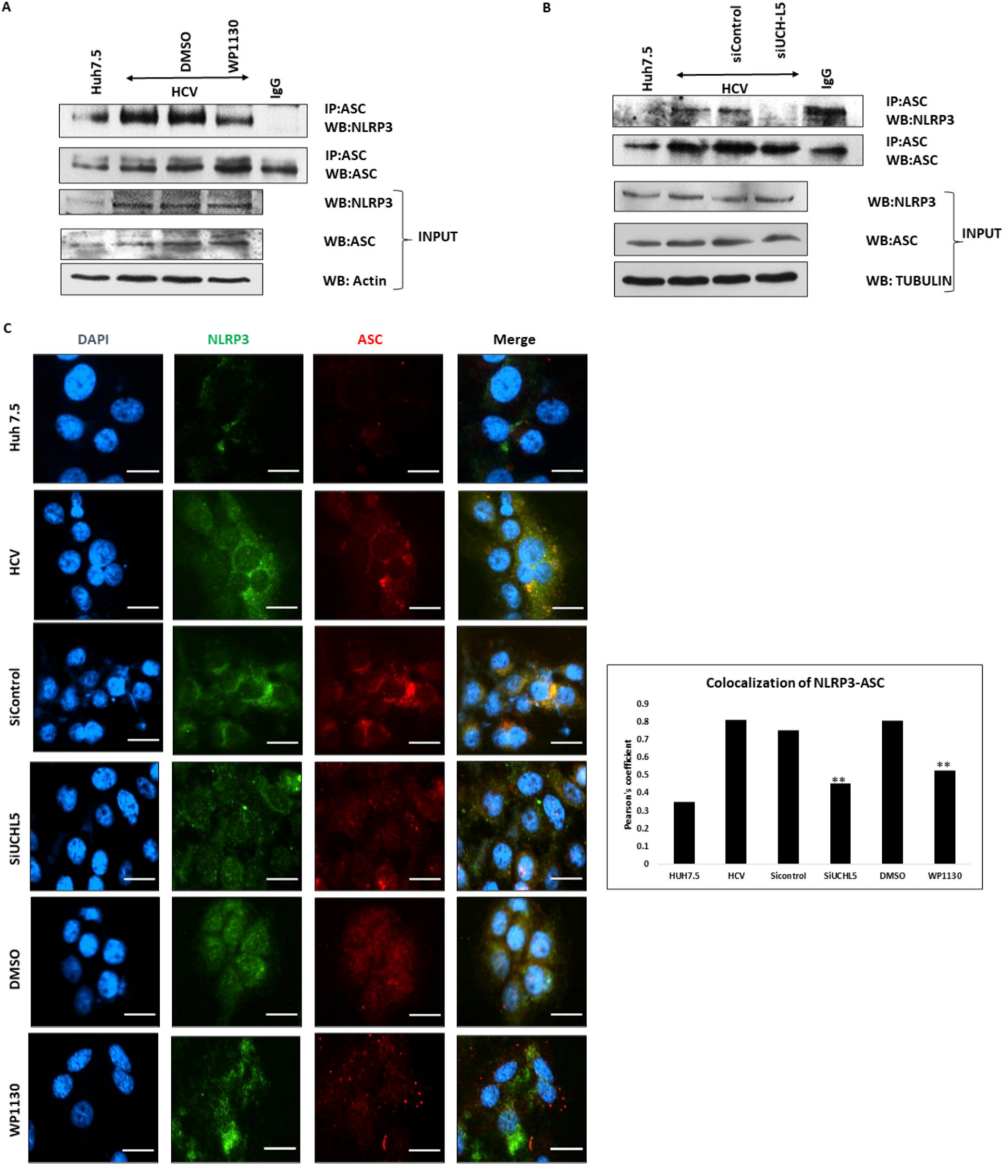
Effect of UCHL5 in NLRP3 inflammasome formation during HCV infection. (A) Huh7.5 (mock) and HCV-infected cells 7 days postinfection (p.i.) were treated with WP1130 (5 μM) for 4 h and lysed in NP-40 lysis buffer. ASC was immunoprecipitated using anti-ASC antibody and Western blotted for NLRP3 and ASC. Input samples were blotted for NLRP3, ACS, and actin. (B) HCV-infected cells were transfected with control or UCHL5 siRNA at 4 days p.i. using electroporation and were lysed 7 days p.i. in NP-40 lysis buffer. ASC was IP using anti-ASC antibody and Western blotted for NLRP3 and ASC. Input samples were blotted for NLRP3, ASC, and tubulin. Representative images are presented from three independent experiments for panels A and B. (C) Mock, HCV-infected, and HCV-infected samples treated with WP1130 (5 μM) or transfected with siUCHL5 were fixed using 4% paraformaldehyde. The cells were washed with phosphate-buffered saline (PBS) and were permeabilized using 0.2% Triton X-100. The cells were washed and blocked using Image-iT FX signal enhancer for 20 min. The cells were then incubated for 2 h using primary antibody for NLRP3 and ASC. After incubation with primary antibody, the cells were washed again and incubated for 2 h with Alexa Fluor 488 and Alexa Fluor 594 secondary antibody for NLRP3 and ASC, respectively. After washing with PBS, the cells were mounted using mounting medium containing DAPI and imaged using a Nikon i80 microscope (scale bars are 20 μm). Mean Pearson’s coefficients were calculated using ImageJ for three fields for each sample containing approximately 50 cells/field. Error bars are the standard deviation from the mean, and significance (*P* < 0.05 [**]) was calculated using a student’s *t* test. Similar results were obtained in two biological replicates.

Inhibition of DUB activity with WP1130 and silencing of UCHL5 increased ubiquitination of NLRP3. To determine if the changes in NLRP3 ubiquitination affected inflammasome assembly and activation, inhibitor-treated cells or UCHL5-silenced cells were examined via immunoprecipitation and immunofluorescence (IFA). Decreased interaction between ASC and NLRP3 via immunoprecipitation was observed in WP1130 inhibitor-treated and UCHL5-silenced cells (Fig. 4A and B). Less colocalization between NLRP3 and ASC was observed in inhibitor-treated and UCHL5-silenced cells by IFA as calculated by Pearson’s coefficient (Fig. 4C and 4D). This suggests that ubiquitination of NLRP3 negatively regulates inflammasome assembly.

### Effect of UCHL5 on NLRP3 inflammasome activation.

**FIG 5:**
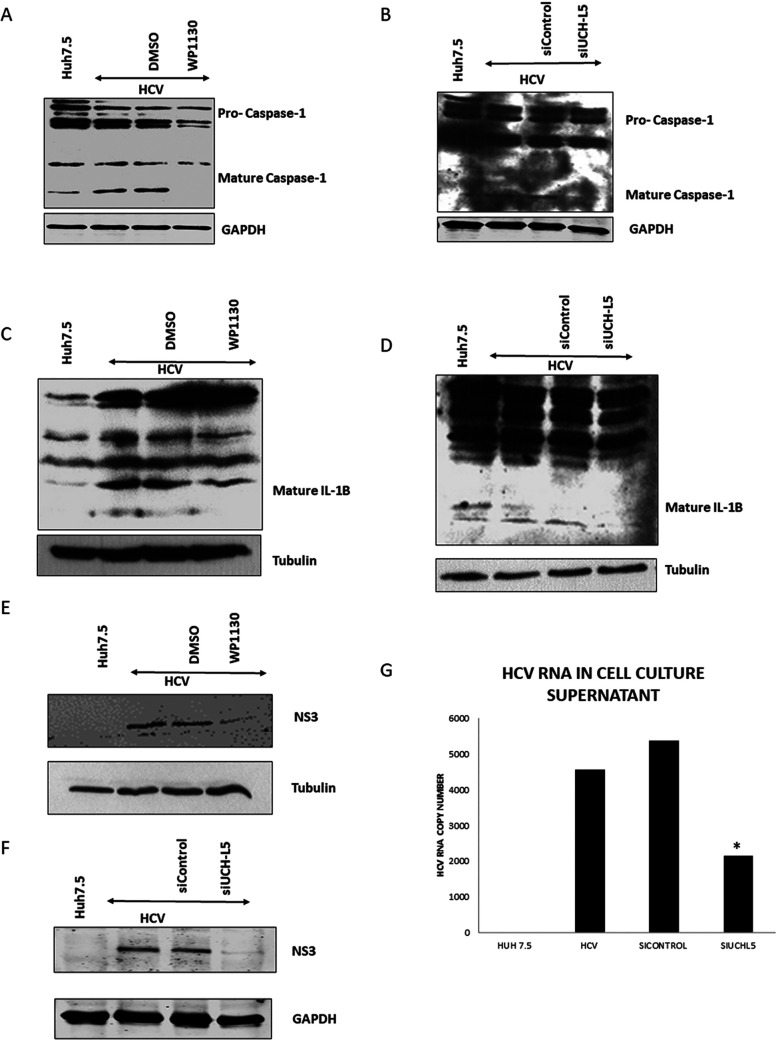
NLRP3 inflammasome activity assessment in the absence of UCHL5 in HCV-infected cells. (A) Huh7.5 (mock) and HCV-infected cells were treated with WP1130 (5 μM) for 4 h and lysed in RIPA buffer and Western blotted for caspse-1 and GAPDH. (B) UCHL5 siRNA was transfected in HCV-infected cells by electroporation at 4 days p.i., and at day 7 p.i., the cells were harvested and lysed using RIPA buffer and Western blotted for caspase-1 and GAPDH. (C and D) HCV-infected cells were either treated with WP1130 (5 μM) for 4 h on day 7 p.i. and harvested or transfected with siUCHL5 at day 4 p.i. and harvested at day 7 p.i. Cells were harvested using RIPA buffer and subjected to Western blotting analyses using anti-IL-1β antibody actin and tubulin. (E and F) HCV-infected cells treated or transfected as in panels C and D but were Western blotted for viral protein HCV-NS3 and tubulin or GAPDH. (G) Cell culture supernatants were collected from control, infected, and infected-siRNA transfected cells as in panels B and D. RNA was isolated from equal volumes of cell culture supernatants, and RT-PCR was performed to quantify the HCV RNA. Error bars are the standard deviation from the mean and significance (*P* < 0.05 [*]) was calculated using a student’s T-test. Representative images and data are presented from three independent experiments.

To determine if inhibition of DUB activity also regulates the activation of the NLRP3 inflammasome, DUB-inhibited or -silenced cells were assayed for the maturity of caspase-1 and IL-1β by Western blotting. Active caspase-1 was decreased in HCV-infected cells that were treated with WP1130 or silenced for UCHL5 (Fig. 5A and B). Active caspase-1 cleaves IL-1β to its active form, and less cleaved IL-1β was also observed in WP1130- and UCHL5-silenced cells (Fig. 5C and 5D). These results indicate that deubiquitination of NLRP3 by UCHL5 is important for inflammasome assembly and activation.

Finally, to determine if the failure to induce and activate the NLRP3 inflammasome via deubiquitination has an effect on HCV replication, Western blotting was performed. Inhibitor-treated and UCHL5-silenced cells were assayed for NS3 expression. Significantly less NS3 was expressed in the inhibitor-treated or DUB-silenced cells (Fig. 5E and F). Release of HCV was also analyzed through quantitative real-time RT-PCR for HCV RNA in the cell culture supernatant. In UCHL5-silenced cells, less HCV RNA was present in the culture supernatants (Fig. 5G). Overall, our experiments have shown that NLRP3 is deubiquitinated during HCV infection, and this deubiquitination is mediated by deubiquitinase enzyme UCHL5. UCHL5 is enzymatically active, and inhibition of UCHL5 by inhibitor WP1130 as well as silencing UCHL5 using siRNA has resulted in inhibition in NLRP3 inflammasome formation and activation as well as inhibition of HCV virus production in infected hepatocytes.

## DISCUSSION

Inflammation of the liver during the course of HCV chronic infection has been observed in patients. A number of HCV-triggered inflammatory mechanisms have been identified. Kupffer cells, hepatic stellate cells, and monocytes/macrophages in the liver have been shown to produce a number of cytokines and chemokines, such as IL-1β, IL-6, IL-8, and tumor necrosis factor alpha (TNF-α) (20–22). In addition, T helper 1 (Th1) and Th2 lymphocytes produce cytokines IL-2, IL-4, and IL-6 in patients with chronic HCV (23). However, the role of hepatocytes, which are the major sites of HCV replication, in inducing inflammation has not been fully elucidated. Our study establishes that the NLRP3 inflammasome complex, which can induce proinflammatory cytokine IL-1β production, is assembled and activated in human hepatoma cells. Indeed, we observed cleaved IL-1β by Western blotting, but we failed to observe IL-1β via enzyme-linked immunosorbent assay (ELISA) in the supernatant or concentrated supernatant of HCV-infected hepatocytes (data not shown). This is consistent with a number of studies that have also reported that they were not able to observe IL-1β secretion from the hepatocytes when compared to myeloid and lymphocytes (24, 25). Future experiments may determine whether the cleaved IL-1β is not secreted, is secreted but not to physiologically relevant levels, or is secreted and helps to induce inflammation within the microenvironment of the infected hepatocytes.

The mechanism by which the NLRP3 inflammasome is activated in HCV-infected hepatocytes has not been elucidated. HCV infection can lead to endoplasmic reticulum (ER) stress, increased intracellular reactive oxygen species (ROS), and activation of NF-κB, AP-1, and STAT3 (26, 27). Studies from Bauernfeind et al. (28) and Boaru et al. (29) show that NLRP3 expression is activated by NF-κB signaling, including hepatocytes. Since NF-κB is activated during HCV infection (30), this may be a licensing or priming signal for NLRP3 inflammasome assembly and activation. In addition to the priming signal, a second signal is required to complete activation of the NLRP3 inflammasome. In hepatocytes, such a secondary signal has not been identified but could include HCV PAMPs or ATP. Further studies are required to understand the specific signals that trigger NLRP3 inflammasome activation.

Our studies identify a role of deubiquitination by UCHL5 in NLRP3-ASC interaction and inflammasome formation. UCHL5, an enzyme from the UCH family of DUBs, is known to prevent degradation of poorly ubiquitinated proteins by removing the polyubiquitin chains (31). In addition, increasing evidence indicates a role for UCHL5 in infection. Our data coincides with studies by Qu et al. (32) as well as Kummari et al. (33) that have shown that UCHL5 is required for NLRP3 inflammasome activation in mycobacterial and salmonella infections, respectively. Zhang et al. also showed that inhibition of UCHL5 blocked TNF-α and IL-6 induction in lipopolysaccharide (LPS)-treated THP-1 macrophages (34). Our study indicates that UCHL5 is also important for NLRP3 activation during viral infection.

We did not observe a difference in the expression of UCHL5 during HCV infection and, therefore, attributed the NLRP3 deubiquitination to activation of the enzyme UCHL5 without its induction. Using an activity-based probe (HA-Ub-VS), which binds to active DUB enzymes, we showed that UCHL5 is enzymatically active in HCV-infected cells. Although, we have shown that UCHL5 is activated in HCV-infected cells, the mechanism leading to UCHL5 activation is unknown. One research study suggests that DUBs USP7 and USP47 are activated with known inflammasome activating signals, such as ROS production, K^+^ efflux, and lysosome destabilization, similar to the secondary signal of NLRP3 inflammasome activation (35). It is possible that UCHL5 may also be regulated by similar signals during HCV infection.

Finally, we have shown evidence that UCHL5 is required for some aspect of the HCV life cycle. Decreased UCHL5 or inhibition of UCHL5 led to a decrease in HCV viral protein NS3 and reduction of HCV RNA in the cell culture supernatant. This may be a direct effect of UCHL5 on the ubiquitination of viral proteins or an indirect effect through an activity of the NLRP3 inflammasome. In addition to regulating inflammatory mediators, studies have shown that caspase-1, which is activated by the NLRP3 inflammasome, can regulate as many as 40 other cellular proteins (36). Specifically, a study by Gurcel et al. showed that caspase-1 activation by bacterial pore-forming toxins activates sterol regulatory binding element proteins (SREBPs) (37). Intriguingly SREBPs are the master transcriptional regulators of lipid metabolism proteins. Therefore, the NLRP3 inflammasome could induce lipid biosynthesis through the activation of SREBPs that is required for HCV replication in infected hepatocytes. The connection between HCV replication, SREBP activation, and NLRP3 inflammasome activation will be the subject of future studies.

In conclusion, the current study demonstrates that the NLRP3 inflammasome is activated by deubiquitination by UCHL5 in HCV-infected hepatocytes. Further studies are required to understand how UCHL5 is activated and the role of NLRP3 inflammasome in regard to either proinflammatory pathways or other cellular signaling pathways in these HCV-infected hepatocytes. Understanding the inflammasome activation and role of UCHL5 pathogenesis of liver disease in the HCV-infected liver can help in generating different therapeutic strategies to target underlying liver disease as well as novel strategies to inhibit HCV infection.

## MATERIALS AND METHODS

### Plasmids and reagents.

The plasmid pJ6/JFH-1 containing infectious HCV cDNA (genotype 2a) was obtained from C. Rice (Rockefeller University, NY). All DUB inhibitors WP1130, ML323, NSC 632839, LDN57444, and 1,10-phenanthroline were purchased from LifeSensors.

### Antibodies.

The following antibodies were used throughout the study and according to the manufacturer’s directions: NLRP3, ASC, and caspase-1 (AdipoGen); NLRP3 (Abcam); ASC (MBL); GAPDH (ProSci); actin and β-tubulin (Sigma); total ubiquitin (PD41), USP9x/y, USP5, USP14, and UCHL5 (Santa Cruz Biotechnology); K63-linked ubiquitin antibody (Cell Signaling Technologies); linear ubiquitin (LUB9; LifeSensors); IL-1β (R&D Systems, Novus Biologicals); and HCV NS3 (ViroGen).

### Cell culture and HCV infection system.

The human hepatoma cell line, Huh7.5, (gift from Charles Rice) was used for all of the experiments. Huh7.5 cells were cultured in Dulbecco modified Eagle medium (DMEM) supplemented with 10% fetal bovine serum, 100 units of penicillin/ml, and 100 μg of streptomycin sulfate/ml and incubated at 37°C and in a humidified atmosphere containing 5% CO_2_. For HCV infection, pJ6/JFH-1 was linearized and transcribed using AmpliScribe T7 transcription kit to generate viral RNA *in vitro*. The transcribed RNA was electroporated into the hepatoma cell line Huh7.5 to produce infectious HCV (38). The cell culture supernatant containing HCV was used to infect naive Huh7.5 cells at a multiplicity of infection (MOI) of 1 in all experiments.

### Western blotting and coimmunoprecipitation.

Uninfected Huh7.5 (mock) and HCV-infected cells were lysed by using radioimmunoprecipitation assay (RIPA) buffer (50 mM Tris/HCl [pH 7.5], 150 mM NaCl, 1% NP-40, 0.5% sodium deoxycholate, 0.1% SDS, 1 mM sodium orthovanadate, 1 mM sodium fluoride, 1 mM EDTA, and 10 μl/ml of protease inhibitor cocktail [Thermo Fisher Scientific]). Equal amounts of protein were subjected to SDS-PAGE and transferred onto a nitrocellulose membrane (Thermo Fisher Scientific). The membranes were blocked using 5% nonfat dry milk in Tris-buffered saline (TBS) (20 mM Tris/HCl [pH 7.5] and 150 mM NaCl) for 1 h at room temperature. The membranes were probed with primary antibodies overnight at 4°C followed by washing of the membranes using TBST (TBS plus 1% Tween 20) three times for 10 min each. After washing, the membranes were incubated with secondary antibodies for 2 h followed by washing with TBST. The immunoblots were visualized using either the LI-COR Odyssey or chemiluminescent exposure of X-ray film.

For immunoprecipitations, uninfected and HCV-infected cells were lysed using NP-40 lysis buffer (150 mM NaCl, 1% NP-40, 50 mM Tris, pH 8.0, 2 mM EDTA, 10% glycerol, and 10 μl/ml of protease inhibitor cocktail). Equal amounts of protein were diluted in NP-40 lysis buffer and precleared with protein G Sepharose beads for 2 h at 4°C. After preclearing, the samples were centrifuged at 1,000 rpm for 5 min, and supernatants were transferred to new centrifuge tubes. Two micrograms of immunoprecipitating antibody were added to the samples and incubated with gentle rotation over night at 4°C. The next day, 50 μl of the protein Sepharose G beads was added to samples to allow antibody to bind to the beads and incubated for 2 h at 4°C with gentle rotation. After incubation with beads, the samples were centrifuged and beads were retained. The beads were washed 3 times with the NP-40 lysis buffer, and bound proteins were eluted with 2× SDS sample buffer. The eluted samples were subjected to Western blotting to test for interaction of target proteins. Control immunoprecipitations were performed with nonspecific IgG antibody to determine nonspecific interactions.

### Detection to ubiquitinated proteins.

NLRP3, ASC, and caspase-1 ubiquitination was determined by following protocol described by Juliana et al. (39). Briefly, cells were lysed in denaturation lysis buffer (50 mM Tris/HCl [pH 7.5], 150 mM NaCl, 1% SDS, 10 mM *N*-ethylmaleimide [NEM], and 10 μM protease inhibitor cocktail). The cellular lysates were boiled for 10 min at 100°C, and equal amounts of protein lysates were diluted with 9 volumes using ubiquitination binding buffer (50 mM Tris/HCl [pH 7.5], 150 mM NaCl, 0.5% NP-40, and 10 mM NEM). NLRP3 and ASC were immunoprecipitated using anti-NLRP3 or anti-ASC antibody and Protein G Sepharose beads (GE) for 4 h at 4°C. Following incubation, the beads were washed with ubiquitination binding buffer, and immunoprecipitated proteins were eluted from the beads using 2× SDS sample buffer. The eluted samples were Western blotted with antiubiquitin and anti-NLRP3 antibody.

### Immunofluorescence assay and proximity ligation assay.

Mock and HCV-infected cells were washed and fixed using 4% paraformaldehyde for 10 min at room temperature. Fixed cells were permeabilized for 5 min using 0.2% Triton X-100 and blocked for 20 min using Image-iT FX signal enhancer (Thermo Fisher Scientific). The cells were then incubated with specific primary antibody for 2 h at room temperature. The cells were then washed and incubated with Alexa Fluor-labeled secondary antibodies (Invitrogen) for 2 h. Stained cells were mounted with antifade reagent containing DAPI (4′,6-diamidino-2-phenylindole) (Invitrogen) and observed using a Nikon 80i fluorescence microscope. To quantify the colocalization between NLRP3 and ASC, Pearson’s coefficient was calculated using ImageJ software. The mean colocalization was calculated from three image fields containing 50 cells for each sample.

Proximity ligation assay (PLA) is used to detect proteins that are in close proximity, less than 40 nm. To perform the assay, we used the Duolink PLA kit (Sigma) according to manufacturer’s instructions. Briefly, mock and HCV-infected samples were washed, fixed, and permeabilized as described above. Duolink blocking buffer was used to block the cells for 30 min at 37°C. After blocking, the cells were washed and incubated for 1 h at 37°C with target specific antibodies, which were diluted in Duolink antibody diluent. Afterword, the cells were incubated with PLA probes (PLUS and MINUS), which was followed by hybridization, ligation, and amplification to detect either red or green dots using a Nikon i80 microscope. To perform double PLA, we first completed the reaction using target antibodies for the first complex and used Duolink red detection reagent. The second reaction was performed after washing, blocking the cells with Duolink blocking buffer, and using target antibodies for second complex detection, for which Duolink green detection reagent was used.

### Knockdown of target gene using siRNA.

The expression of DUBs UCHL5, USP5, and USP14 were inhibited with siRNA purchased from Santa Cruz Biotechnology. The siRNA was supplied as a pool of five to six siRNA composed of 19 to 25 nt in length. As a control, a nontargeting (NT) siRNA was used. Briefly, siRNA was electroporated into HCV-infected cells (day 4 postinfection) using a Neon transfection system (Invitrogen). The cells were trypsinized, centrifuged, and resuspended in electroporation buffer with 10 μl of either NT siRNA or DUB siRNA. The mixture of cells and siRNA was subjected to an electric shock at 1,100 V, 1 pulse, 30 ms, after which the cells were cultured in DMEM and incubated for 48 to 72 h. Postelectroporation, the cells were harvested depending on the experiment in either RIPA buffer, NP-40 lysis buffer, or ubiquitination lysis buffer and analyzed accordingly.

### Detection of DUB activity.

Mock and HCV-infected cells were lysed in buffer containing 0.1% NP-40, 150 mM NaCl, 20 mM CaCl_2_, and 50 mM Tris/HCl (pH 7.4). Equal amounts of protein from mock and HCV-infected lysates were incubated with 1 μM HA-Ub-VS probe (Boston Biochemical) for 1 h at 37°C. Following probe reaction, 6× SDS loading dye was added, and samples were heated at 95°C for 5 min. The samples were then subjected to Western blotting analysis and immunoblotted for specific DUBs or probe (via hemagglutination [HA] tag).

### Quantitative real-time RT-PCR.

To quantify the HCV released from the cells, cell culture supernatants were collected from mock, HCV-infected, and HCV-infected siRNA-transfected samples. Equal volumes of cell culture supernatant (500 μl/sample) were used to isolated RNA using the RNeasy minikit (Qiagen). HCV-RNA copy numbers were determined as by real-time RT-PCR using ABI PRISM 7500 sequence detector (Applied Biosystems). HCV-specific primers FWD- (5′-CGGGAGAGCCATAGTGG-3′) and REV- (5′-AGTACCACAAGGTTTCG-3′) were used along with an HCV-specific 6-FAM-CTGCGGAACCGGTGAGTACAC-TAMRA probe. Reactions were performed in triplicate and in a 25-μl mix using TaqMan Fast Virus 1-step master mix. Reactions were carried out in the following conditions: 5 min at 50°C, 20 s at 95°C, and 44 cycles of 20 s at 95°C and 1 min at 62°C. Copy numbers were determined by comparison to copy number standards ranging from 10^1^ to 10^6^ copies/μg of RNA.
